# B-cell modulators in immunoglobulin A nephropathy

**DOI:** 10.3389/fimmu.2026.1843207

**Published:** 2026-10-08

**Authors:** Arun Rajasekaran, Jan Novak, Dana V. Rizk

**Affiliations:** 1 Renal Medicine Associates, Albuquerque, NM, United States; 2 Department of Microbiology, University of Alabama at Birmingham, Heersink School of Medicine, Birmingham, AL, United States; 3 Division of Nephrology, Department of Medicine, University of Alabama at Birmingham, Heersink School of Medicine, Birmingham, AL, United States

**Keywords:** APRIL, BAFF, B-cell targeting, CD38, galactose-deficient IgA1, IgA nephropathy, plasma-cell depletion

## Abstract

Immunoglobulin A nephropathy (IgAN), defined by mesangial immune-complex deposition enriched for galactose-deficient IgA1 (Gd-IgA1), is the most prevalent biopsy-confirmed primary glomerulonephritis worldwide. Advances in mucosal immunobiology and genetics have underscored the role of dysregulated B-cell activity, increased Gd-IgA1 production, and corresponding Gd-IgA1–specific autoantibodies in the pathogenesis of IgAN. Therapeutic strategies targeting the mucosal immune system, particularly pathways involved in B-cell survival and differentiation, offer a specific approach to disease modification based on our understanding of its pathogenesis. Emerging therapies, including agents targeting gut-associated lymphoid tissue, antagonists of B-cell activating factor (BAFF) and/or a proliferation-inducing ligand (APRIL), and B-cell depletion therapies like anti-CD38 monoclonal antibodies, have demonstrated promising positive results across multiple clinical trials at various stages of development. In this review, we highlight targeting the underlying immunology of IgAN pathogenesis and shed light on newly emerging B-cell modulators for the treatment of the disease.

## Introduction

Immunoglobulin A (IgA) nephropathy (IgAN) is the most common primary glomerular disease globally and remains a leading cause of chronic kidney disease (CKD) and kidney failure (1, 2). It is characterized by IgA-(co)dominant glomerular immune-complex deposits and is routinely associated with both mesangial expansion and cellular proliferation seen by light microscopy in kidney biopsies (3). Clinical presentation is highly variable, spanning from incidental microscopic hematuria with relatively preserved kidney function to rapidly progressive glomerulonephritis (although the latter is rare) (1). In adults, IgAN commonly presents as CKD with sub-nephrotic proteinuria and hematuria, while the hallmark visible hematuria accompanying mucosal infections is seen mostly in children. At the time of diagnosis, over 50% of patients have CKD stage 3 or beyond, indicating significant nephron loss (4, 5). Prevalence differs across ethnic and racial groups, with the highest rates observed in individuals of East Asian descent, followed by Caucasians, and exceedingly low rates seen among those of sub-Saharan African ancestry (6). The incidence of IgAN is estimated at 2–10 per 100,000 persons annually, and peaks during the second and third decades of life (7, 8). Approximately 30-40% of patients progress to kidney failure within 10 years of diagnosis, and this proportion rises to more than 50% over 10–15 years (4, 9). Therefore, efforts for early diagnosis of IgAN need to be expanded and a change in our current management of the disease is of critical importance.

Proteinuria is a key predictor of kidney survival and overall prognosis in IgAN. Earlier data had shown that every 1 g/day increase above 1 g/day pf proteinuria was associated with a 10- to 25-fold faster decline in kidney function (10). More contemporaneous data from the United Kingdom National Registry of Rare Kidney Diseases demonstrated that even a urine protein-to-creatinine ratio (UPCR) level < 0.88 g/g was associated with a higher likelihood of progression to kidney failure within 10 years of biopsy diagnosis (4).

As such, the updated 2025 Kidney Disease: Improving Global Outcomes (KDIGO) treatment guidelines recommend that the aim of treatment in IgAN should target proteinuria of < 0.5 g/day and ideally < 0.3 g/day (11, 12). In some patients with extensive kidney scarring, these thresholds may not be achieved without the potential utilization of multitargeted therapies. Beyond proteinuria, complete remission in IgAN should include estimated glomerular filtration rate (eGFR) annual losses not exceeding 1 mL/min/year in adults (11). In addition, the above guideline for the management of IgAN at risk of progressive kidney function loss recommends managing the IgAN specific drivers for nephron loss by initiation of treatments that prevent or reduce production of pathogenic IgA and the associated immune-complex formation along with halting downstream kidney injury and these should be considered simultaneously with foundational CKD management approaches to manage the consequences of existing IgAN-induced nephron loss (13).

The elucidation of disease pathogenesis over the past five decades since the original description of IgAN have highlighted the critical role of B-cells and plasma cells in the onset and maintenance of the disease. At the center are immunologic abnormalities within the mucosa-associated lymphoid tissue (MALT), which lead to excessive production of galactose-deficient IgA1 (Gd-IgA1) glycoforms that enter the systemic circulation (14). The role of cytokines, particularly a proliferation-inducing ligand (APRIL) and B-cell activating factor (BAFF), in promoting B-cell maturation, differentiation, and survival has paved the way for new therapeutic approaches (15–17). Building upon these foundational pathways, numerous clinical trials targeting mucosal and other B-cell pathways have contributed to the paradigm shift we are witnessing in the IgAN therapeutic landscape.

## Pathogenesis of IgAN: the four-hit hypothesis

The pathogenesis of IgAN is believed to occur through sequential pathogenic “hits” (**Figure 1**) (18). First (hit 1), there is an increase in circulating IgA1 with reduced content of galactose in the hinge region of Gd-IgA1 (19–21). Subsequently, autoantibodies of the IgG or IgA isotypes bind Gd-IgA1 with exposed *N*-acetylgalactosamine residues (hit 2) (22, 23). The IgG isotype seems to play a more important role in disease pathogenesis (24). This leads to the development of circulating immune complexes to which additional proteins are attached (hit 3) (25, 26). Some of these nephritogenic immune complexes deposit within the glomeruli, where they activate mesangial cells and induce cellular proliferation and overproduction of cytokines and profibrotic mediators, that lead to podocyte injury and disruption of the glomerular filtration barrier (hit 4) (18). Complement activation likely participates at the level of circulating immune complexes as well as at the level of glomerular immune-complex deposits (26–29). These processes ultimately lead to hematuria, proteinuria, and glomerular as well as tubulointerstitial injury, resulting in the progressive decline in kidney function, all known features of IgAN.

**Figure 1:**
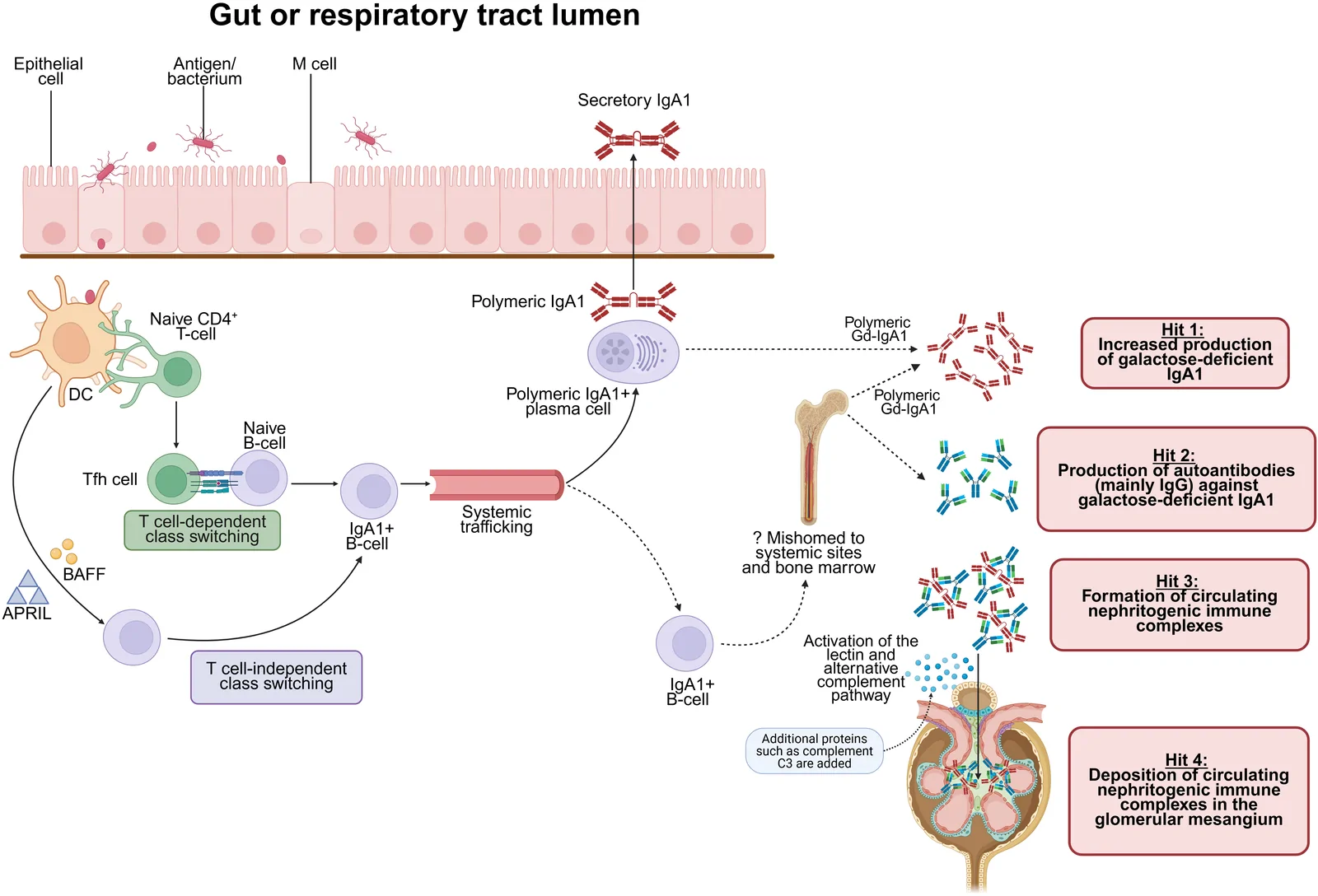
Mucosal B-cell production of galactose-deficient IgA1 (Gd-IgA1) in immunoglobulin A nephropathy (IgAN) and the “Four-hit” hypothesis for the pathogenesis of IgAN. Polymeric immunoglobulin A (IgA) is predominantly produced in mucosa-associated lymphoid tissues, with the gut and respiratory tract representing major sites of IgA production. Mucosal B-cell IgA responses can be initiated through both T-cell–dependent and T-cell–independent pathways. Following antigen uptake by intestinal M cells and dendritic cells (DCs), naïve T cells are activated and differentiate into T follicular helper (Tfh) cells. Tfh cells subsequently interact with naïve IgM- and IgD-expressing B cells, promoting class-switch recombination and differentiation toward IgA-producing B cells within the mucosal environment. Dendritic cells (DCs) can also secrete B-cell activating factor (BAFF) and a proliferation-inducing ligand (APRIL), which facilitate T-cell–independent class-switch recombination toward IgA-producing B cells. These processes ultimately generate IgA-producing B cells that mature and migrate through the lymphatic system to effector sites. In IgA nephropathy (IgAN), some cells produce Gd-IgA1, leading to elevated circulating levels of Gd-IgA1. This process may be potentially because of a “spillover” from mucosal tissues or aberrant homing of Gd-IgA1–producing B cells to systemic compartments, such as the bone marrow. Elevated circulating Gd-IgA1 is the first event of the four-hit model. Hit 1: excessive generation of galactose-deficient IgA1 (Gd-IgA1), which enters the circulation from mucosal or possibly systemic sources. Hit 2: generation of immunoglobulin G (IgG) autoantibodies that recognize Gd-IgA1. Hit 3: formation of nephritogenic IgG-Gd-IgA1 immune complexes in the circulation; additional proteins are added, such as complement C3. Hit 4: some of these circulating IgG-Gd-IgA1 immune complexes deposit in the glomerular mesangium, activate mesangial cells to proliferate and overproduce cytokines and chemokines and components of extracellular matrix, leading to progressive kidney injury. Furthermore, alternative and lectin complement pathways can be activated *in situ*, and an influx of monocytes/macrophages from the circulation may further exacerbate glomerular and tubulointerstitial injury. Figure created with www.BioRender.com.

## Mucosal immune system as a major source of Gd-IgA1

Of all human immunoglobulin isotypes, IgA is the most abundantly produced isotype, accounting for approximately two-thirds of the body’s total immunoglobulins (30, 31). Human IgA exists as two subclasses, IgA1 and IgA2, which differ in the length of their heavy-chain hinge regions. These segments provide the antibody with flexibility, enabling the antigen-binding sites to effectively engage their targets (32). More than 90% of IgA is produced by plasma cells located within mucosa-associated lymphoid tissue (MALT), more specifically the gut-associated lymphoid tissue (GALT) and the nasopharynx-associated lymphoid tissue (NALT) (33, 34). Mucosal secretory IgA, present in secretions, is composed of polymeric IgA molecules containing two or more monomers linked by the joining (J) chain, along with a secretory component acquired during transcytosis (35). Most IgA produced at mucosal sites is polymeric, whereas the majority of circulating IgA is monomeric and is produced in the bone marrow (36). The pathogenic IgA1 found in circulating as well as mesangial immune complexes is predominantly polymeric, and poorly galactosylated, suggesting a mucosal origin (19, 20, 37, 38). The so-called synpharyngitic macroscopic hematuria with visible blood in the urine occurring during upper respiratory tract and/or gastrointestinal infections underscores the significance of the mucosal-kidney nexus in IgAN (34, 39). Alterations in the tonsillar and gut microbiome have been reported in IgAN patients compared with individuals with tonsillar hyperplasia and healthy controls (40, 41).

Within the MALT, mucosal antigens are frequently processed by antigen-presenting cells like dendritic cells (DCs) which leads to the activation of naïve B cells and enables class-switch recombination (CSR) to IgA+ B-cells. This occurs through a T-cell-dependent or T-cell-independent process (42, 43). Through this process, naïve B-cells undergo CSR that determines immunoglobulin class, and somatic hypermutations to become antigen-specific class-switched B-cells. Maintenance of normal mucosal immunity depends critically on class switching to IgA production. IgA serves as the first line of defense at mucosal surfaces by eliminating pathogens while also preserving homeostasis with commensal microorganisms. In IgAN, however, disease pathogenesis is closely linked to disruptions in B-cell activation and CSR, which promote increased production of IgA1 overall and, more specifically, Gd-IgA1 (16, 44, 45).

Accumulated data indicates that production of Gd-IgA1 is genetically determined and its production is further affected by various cytokines and growth factors. Development of quantitative lectin ELISA (21) enabled cellular, biochemical, and genetic studies of IgA1 *O*-glycosylation pathways and identification of elements involved in production of Gd-IgA1 (46–52). These studies revealed dysregulated expression of key enzymes involved in *O*-glycosylation of IgA1 in IgA1-producing cells, specifically reduced expression of C1GalT1 and its molecular chaperon Cosmc and elevated expression of ST6GalNAc2. Moreover, cytokines of IL-6 family, including IL-6 and leukemia inhibitory factor (LIF) enhance the production of Gd-IgA1. These two cytokines further dysregulate expression of C1GalT1, Cosmc, and ST6GalNAc2 and consequently increase production of Gd-IgA1. These cytokines affect mainly cells derived from IgAN patients but not cells from healthy controls (47–49).

B-cell activating factor (BAFF), also known as B-lymphocyte stimulator, and a proliferation-inducing ligand (APRIL), both members of the tumor necrosis factor (TNF) superfamily, play central roles in promoting both T-cell-dependent and T-cell-independent CSR, thereby driving the generation of IgA+ B-cells (16, 42, 43, 53–55). The IgA+ B-cells subsequently exit MALT and migrate to effector sites through the lymphatic system and bloodstream. In IgAN, it has been hypothesized that altered homing causes mucosal-derived IgA+ plasma cells to be mis-trafficked to systemic sites, such as the bone marrow, resulting in the production of “mucosal-type” Gd-IgA1 (*i.e.*, polymeric Gd-IgA1) in the systemic circulation (56–58). Furthermore, a recent paper showed that a glycosylation initiation enzyme, GalNAc-T14, engages in regulating homing of B-cells (59). Other work has postulated the possible role for reverse apical-to-basolateral retro-transcytosis of Gd-IgA1 from the gut lumen or trafficking of IgA+ B-cells to the kidneys in IgAN pathogenesis (60, 61). Follow-up studies will need to assess the relevance of these findings to human disease. An important observation is that elevated circulating levels of Gd-IgA1 alone are not sufficient to cause IgAN, as some first-degree relatives of patients with IgAN were found to have increased systemic Gd-IgA1 levels but remained disease free during long follow-up observation (21, 62). The onset of kidney injury in IgAN requires the formation of autoantibodies and subsequently immune complexes that also contain other proteins such as complement C3 (23, 63, 64).

## Role of B-cells in IgAN pathophysiology and implications for treatment

B-cells are responsible for producing Gd-IgA1 (the main autoantigen) and IgG autoantibodies that are specific for Gd-IgA1, the two pivotal components driving formation of circulating immune complexes in IgAN (39, 42). BAFF and APRIL are two cytokines in the TNF family that share approximately 30% homology and play essential roles in B-cell differentiation. Notably, genome-wide association studies have identified several susceptibility genes for IgAN, including *TNFSF13* (located on chromosome 17p13) and *TNFSFR13B*, which encode APRIL and its receptor, TACI, respectively (65). Both BAFF and APRIL bind, with differing affinities, to two shared receptors: B-cell maturation antigen (BCMA; encoded by the *TNFRSF17* gene) and TACI (**Figure 2**) (54, 55). BCMA is predominantly expressed on plasma cells, whereas TACI is primarily found on mature B-cells and activated plasma cells. The BAFF receptor (BAFF-R) is highly specific for BAFF and is expressed on the surface of most peripheral B-cell subsets, except for plasma cells and germinal center centroblasts (54, 55, 66, 67). In addition, BAFF-R is expressed on human mesangial cells and tubular epithelial cells (TECs) (67–69). BAFF increases BAFF-R expression in human TECs, thereby sustaining an autocrine signaling loop (68). APRIL binds strongly to BCMA and with lower affinity to TACI, whereas BAFF shows stronger binding to TACI and weaker affinity for BCMA (70). In addition, APRIL can bind to heparan sulfate proteoglycans in the extracellular matrix or on the cell surface, including those on plasma cells, thereby enhancing its local concentration and biological activity (71). B-cells play a major role in the adaptive immune system. They undergo a tightly regulated process of differentiation, maturation, and selection to be able to produce antibodies in response to antigens (72). B-cells can differentiate into short-lived plasmablasts or plasma cells, as well as long-lived plasma cells, all of which are important to produce immunoglobulins, including IgA (55, 73, 74). Memory B-cells and plasma cells also play key roles in maintaining immunologic memory (**Figure 3**) (75).

**Figure 2:**
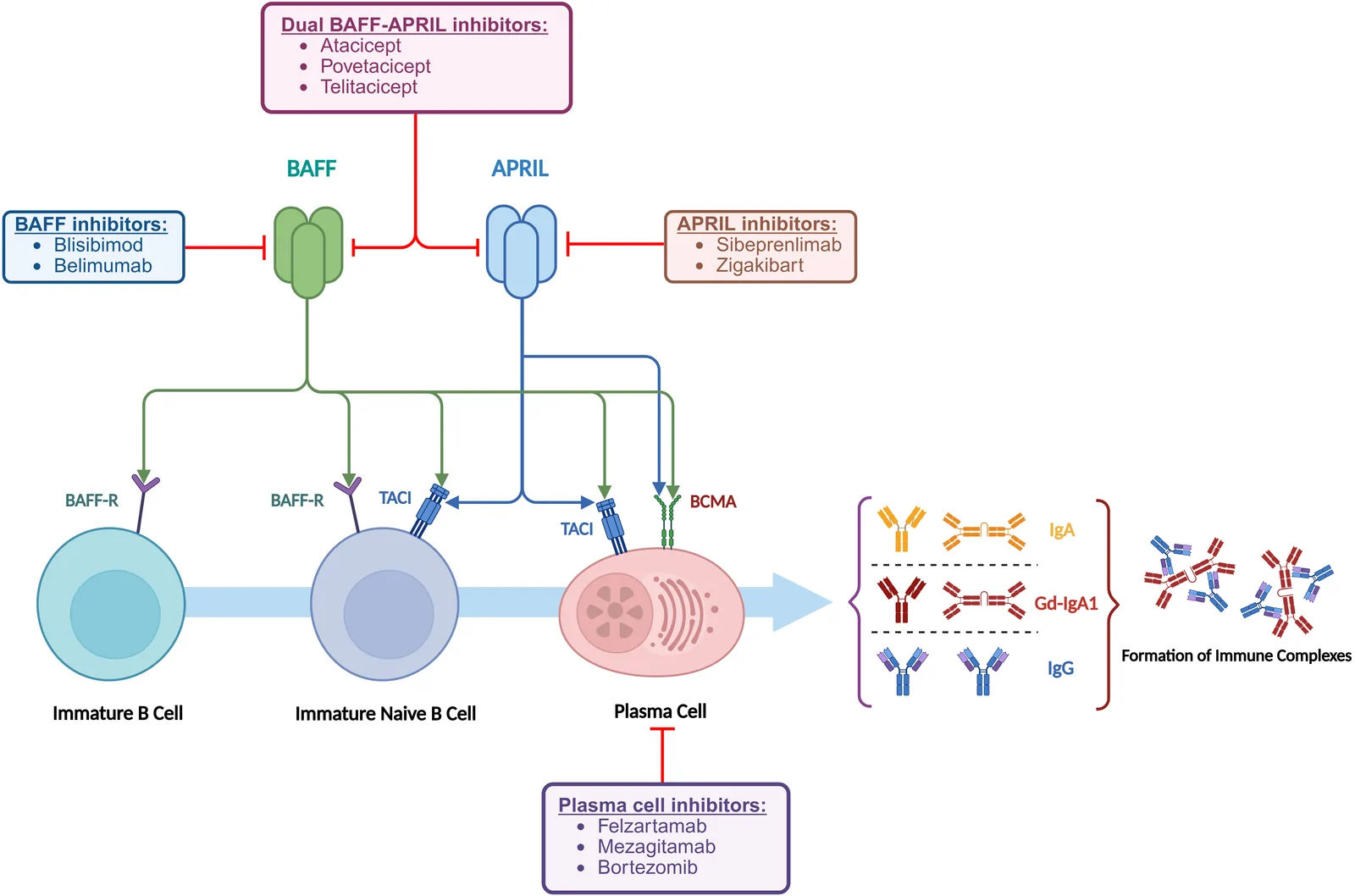
Overview of BAFF, APRIL, and associated B-cell-directed treatments in IgAN. B-cell activating factor (BAFF) and a proliferation-inducing ligand (APRIL) are cytokine members of the tumor necrosis factor superfamily. Both interact with two shared receptors: B-cell maturation antigen (BCMA) and transmembrane activator and calcium-modulator and cyclophilin ligand interactor (TACI). BCMA is expressed predominantly on plasma cells, whereas TACI is primarily found on mature B-cells and plasma cells. A third receptor, BAFF receptor (BAFF-R), which is specific to BAFF, is mainly expressed on immature and mature naïve B-cells. In IgAN, BAFF and APRIL play pivotal roles in the interplay and crosstalk between gut mucosal hyperresponsiveness, B-cell and plasma-cell activation, and the resulting overproduction of Gd-IgA1 and autoantibodies specific for Gd-IgA1, ultimately leading to immune complex formation. Not shown in this figure is the interaction of APRIL with heparan sulfate proteoglycans found on the surface of B-cells, especially plasma cells and memory B-cells, but also other cells such as dendritic cells, macrophages, and monocytes. This interaction enhances APRIL’s local concentrations and downstream effects. Therapies that inhibit BAFF, APRIL, or both, and plasma-cells targeted therapies are also depicted. APRIL, A proliferation-inducing ligand; BAFF, B-cell activating factor; BAFF-R, B-cell activating factor receptor; BCMA, B-cell maturation antigen; Gd-IgA1, Galactose deficient immunoglobulin A1; HSPG, Heparan sulfate proteoglycan; IgA, Immunoglobulin A; IgG, Immunoglobulin G; IgAN, Immunoglobulin A nephropathy; TACI, Transmembrane activator and calcium-modulator and cyclophilin ligand interactor. Figure created with www.BioRender.com.

**Figure 3:**
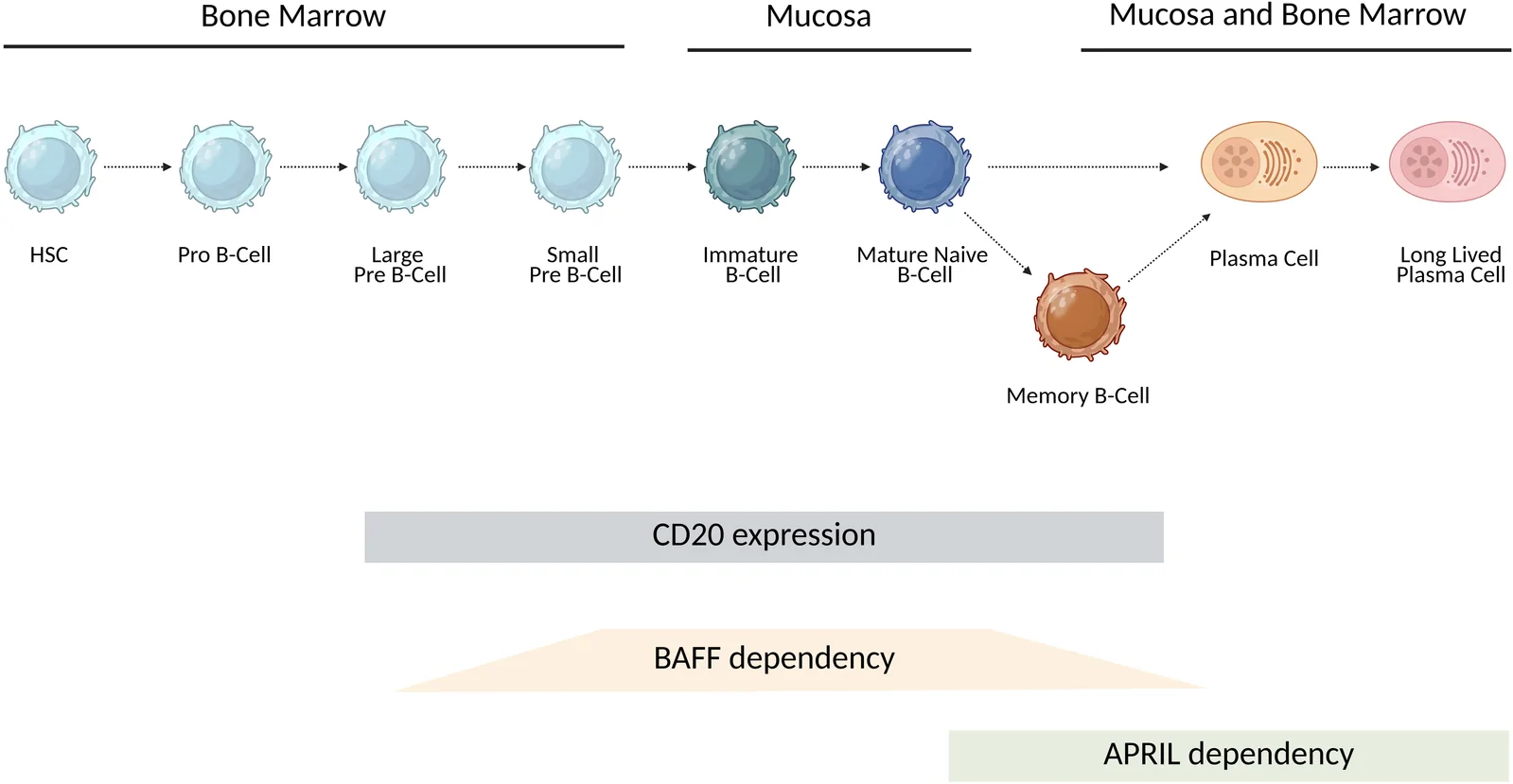
Various stages of B-cell maturation. B-cells undergo a sequential process of selection and maturation and play a pivotal role in the adaptive immune system by generating antibodies as a response to foreign antigens. On leaving the bone marrow, B-cells migrate to peripheral and secondary lymphoid organs, including mucosa-associated lymphoid tissue. B-cells can differentiate into short-lived plasmablasts or plasma cells, as well as long-lived plasma cells, all of which are responsible for producing immunoglobulins, including IgA. CD19 is a pan-B-cell surface marker, CD20 expression is lost as B-cells mature into plasmablasts and plasma cells; whereas CD38 is highly expressed on plasmablasts and plasma cells. APRIL, A proliferation-inducing ligand; BAFF, B-cell activating factor; BAFF-R; HSC, Hematopoietic stem cells; IgA, Immunoglobulin A. Figure created with www.BioRender.com.

## Role of BAFF and APRIL in B-cell and plasma cell homeostasis

BAFF is a key regulator of B-cell maturation and is essential for the activation, differentiation, and survival of peripheral B-cells. Its interaction with its principal receptor, BAFF-R, is critical for maintaining B-cell homeostasis (76). B-cell activation requires antigen presentation together with a co-stimulatory signal and can occur through either T cell–dependent or T cell–independent mechanisms (42, 43, 77). Microbial antigens that contain pathogen-associated molecular patterns (PAMPs) likely trigger the release of BAFF and APRIL from antigen-presenting cells, such as dendritic cells, through engagement of pattern-recognition receptors, including Toll-like receptors (TLRs), on their surface, thereby linking innate and adaptive immune responses (78). BAFF and APRIL subsequently activate B-cells through their receptors BAFF-R, BCMA, and TACI (54, 55). Activation of these receptors accelerates IgA CSR and promotes B-cell survival (54, 78), thereby enhancing microbe-driven IgA B-cell responses at the mucosal sites (79).

BAFF promotes the survival and differentiation of B-cells into long-lived plasma cells that reside in the bone marrow, secondary lymphoid organs, and mucosal tissues (80, 81). These plasma cells can continuously produce antibodies, including Gd-IgA1, without requiring further stimulation. APRIL was initially recognized for its ability to stimulate tumor cell proliferation *in vitro*, but it has since been recognized to play important roles in multiple B-cell functions (55). APRIL is produced by numerous cell types, including myeloid cells (monocytes, macrophages, dendritic cells), activated lymphocytes, and mucosal epithelial cells (55). APRIL is produced by a wide range of cell types, including myeloid cells such as monocytes, macrophages, and dendritic cells, as well as activated lymphocytes and mucosal epithelial cells (55). It plays a key role in B-cell survival and differentiation (55, 73). APRIL promotes IgA production by B-cells. *In vitro* studies have shown that stimulation of B-cells from IgAN patients with APRIL leads to increased production of Gd-IgA1, suggesting a direct role in driving abnormal glycosylation of IgA1 (82).

## Preclinical evidence for the role of BAFF in IgAN

Compared with wild-type mice, transgenic mice overexpressing BAFF (BAFF-Tg) exhibit several notable immunologic alterations, including persistently elevated serum IgA levels (74). This elevated serum IgA is associated with an increased number of IgA+ plasma cells in the gut lamina propria. IgA-containing immune deposits in the glomerular mesangium, resembling those seen in human IgAN were observed, but only in the presence of gut microbiota. These mice also exhibited a marked expansion of the B-cell repertoire, likely contributing to autoimmunity. In addition, BAFF-Tg mice demonstrated significant increase in mature B-cells, spontaneous germinal center formation, autoantibody production, elevated plasma cell numbers in secondary lymphoid organs, increased effector T cells, and immunoglobulin deposition in the kidneys (76, 83).

Using a similar animal model with different experimental conditions, BAFF-Tg mice exhibited increased circulating levels of aberrantly glycosylated polymeric IgA (with altered *N*-glycosylation, as murine IgA lacks *O*-glycans), along with mesangial IgA deposition and expansion of the mesangial matrix. These mice also developed IgA-dependent hematuria and proteinuria. Importantly, the presence of commensal bacteria was required for the development of this IgAN-like phenotype. BAFF-Tg mice raised in sterile, germ-free conditions that prevented gut colonization by commensal bacteria were protected from elevated serum IgA levels, glomerular IgA deposition, expansion of gut IgA+ plasma cells, and the presence of circulating IgA antibodies reactive to commensal bacteria. These features reappeared, however, once commensal bacteria were introduced (79).

Currie et al. characterized immune responses to mucosal microbiota in BAFF-Tg mice and found that nasal exposure to *Neisseria meningitidis* led to elevated circulating bacterium-specific IgA levels, along with significant mesangial matrix expansion and glomerular IgA deposition. Of particular importance, an increase in antigen-specific IgA+ secreting cells was observed in kidney tissue, suggesting that these cells migrated there from a mucosal source after priming and could contribute to local IgA production and further deposition in glomeruli. These observations together suggest that disruption to mucosal microbiota and hyperreactive B-cells primed by elevated levels of BAFF could both likely lead to the IgAN-like phenotype noticed in this model (61). Despite the relevance of BAFF seen in murine models of IgA nephropathy, BAFF inhibition alone has not been shown to significantly affect nephritogenic IgA production or disease progression in these animal models. In a recent study using an IgAN-prone mouse model, treatment with an anti-BAFF antibody failed to reduce circulating levels of aberrantly *N*-glycosylated IgA or IgG-Gd-IgA1 immune complexes. In addition, no improvement in albuminuria or kidney histology features were observed compared with control animals (84). These results suggest that additional ligands, particularly APRIL, may play a role in driving IgAN pathogenesis.

## Preclinical evidence supporting the role of APRIL in IgAN

Experimental studies have shown that microbial exposure can trigger TLR activation, leading to APRIL release from dendritic cells and subsequent Gd-IgA1 production (85). The ddY mouse strain is a model of spontaneous-onset IgAN. Maintaining these mice in a germ-free environment protects them from developing kidney disease (86). However, the intranasal administration of the TLR9-ligand CpG oligodeoxynucleotide, a highly antigenic bacterial cell wall component, could induce an IgAN-like disease phenotype. On the other hand, fecal transplantation of gut bacteria did not induce such pathological changes underscoring the important role of nasopharynx-associated lymphoid tissue in this model (86). *In vitro*, stimulation of dendritic cells with CpG oligodeoxynucleotide led to increased serum APRIL levels, and culture of B-cells in the resulting supernatant was associated with enhanced IgA production (87). CpG oligodeoxynucleotide also enhanced IgA production by murine B-cells, an effect that was significantly reduced in TACI knockout mice (88). Myette et al. assessed the efficacy of APRIL inhibition in the grouped ddY (gddY) mouse model of IgAN. Treatment with a mouse anti-APRIL monoclonal antibody resulted in reduced serum IgA levels and circulating immune complexes, along with decreased glomerular deposition of IgA, IgG, and C3, and a reduction in proteinuria (89).

A study showed that the intestinal microbiota of patients with IgAN had a higher relative abundance of mucin-degrading bacteria (including *Akkermansia muciniphila*) compared with patients with other causes of CKD and with healthy individuals. These bacteria were able to de-glycosylate IgA1 *in vitro* and were associated with increased levels of de-glycosylated IgA1 *in vivo* in a humanized mouse model of IgAN. Following retrotranscytosis of this IgA1 from the luminal surface into the systemic circulation, it has been proposed that IgA1-containing immune complexes may form and subsequently deposit in the murine glomeruli (90). However, it is to be noted that such IgA1 will contain the secretory component, and it is not clear how the findings from the animal experiments are applicable to human IgAN. In fact, both polymeric and monomeric IgA1 molecular forms were found in human biopsy specimens (38), and the secretory component was detected in only a small fraction of IgAN cases (91, 92). Although we have gained important insights about the contributions of BAFF and APRIL to IgAN pathogenesis from the ddY/gddY and BAFF-overexpressing mouse models, their relevance to human disease should be interpreted with caution. Mice express only a single form of IgA that lacks the *O*-glycosylated extended hinge region and therefore resembles mostly human IgA2. To address these issues, humanized mice expressing human IgA1 were developed (93) and a passive mouse model of IgAN was used wherein immune complexes formed with human Gd-IgA1 and human IgG autoantibodies were injected into immunodeficient mice (94, 95).

## Clinical and translational evidence of APRIL and BAFF dysregulation in IgAN

Multiple studies have shown that serum BAFF levels are elevated in patients with IgAN compared with healthy controls (42, 79, 96, 97). In addition, serum BAFF levels have been found to correlate positively with IgA1 levels (96). BAFF has been associated with both the clinical and histologic severity of IgAN, and serum levels of BAFF correlate positively with the extent of mesangial IgA deposition (97), as well as the presence of mesangial hypercellularity, segmental glomerulosclerosis and interstitial fibrosis/tubular atrophy (96). Additionally, elevated circulating BAFF levels have been associated with impaired kidney function (96). Other studies have shown that tonsillar mononuclear cells from patients with IgAN can be induced to express higher levels of BAFF and IgA than cells from non-IgAN controls (98, 99).

Elevated circulating APRIL levels have been reported in multiple cohorts of patients with IgAN compared with healthy controls (42, 79, 82, 100). McCarthy et al. reported elevated serum APRIL levels, along with modest increases in BAFF levels, in some patients with IgAN. APRIL levels were associated with higher serum creatinine and greater proteinuria (79). Zhai et al. demonstrated that serum APRIL levels were elevated in patients with IgAN and correlated with Gd-IgA1 levels as well as with greater clinical severity, such as higher proteinuria and lower eGFR (82). APRIL mRNA expression was increased in peripheral blood B-cells isolated from a subset of patients with IgAN (82). Han et al. reported elevated plasma APRIL levels in 637 patients with IgAN, which were associated with an increased risk of progression to kidney (100). In a Japanese IgAN cohort, increased tonsillar APRIL expression was reported and was associated with higher levels of proteinuria (101). Furthermore, patients who underwent kidney transplantation and had evidence of recurrent IgAN in the allograft, were noted to have a rise in serum APRIL levels at 6 months post-transplant that remained persistently elevated for up to 3 years whereas their serum BAFF levels did not (102).

As noted earlier, genome-wide association studies have identified IgAN-risk alleles at the 17p13 locus encoding *TNFSF13* (gene encoding APRIL) and in the 17p11 locus encoding TACI receptor (*TNFRSF13B*), providing further justification for targeting APRIL and TACI in the treatment of IgAN (65, 100, 103–105). However, it remains unclear whether long-term inhibition of APRIL or BAFF alone in patients with IgAN may lead to compensatory upregulation of the other cytokine. Notably, a rare case of common variable immunodeficiency with complete APRIL deficiency was reported to have elevated circulating BAFF levels (106).

Collectively, the preclinical and clinical data reinforce several principles with direct relevance to IgAN (1): BAFF and APRIL are important drivers of B-cell survival and mucosal IgA CSR (2); modest increases in BAFF/APRIL can shift the B-cell repertoire toward autoreactivity and excess immunoglobulin class-switching (3); TACI and BCMA signaling are central for the persistence of long-lived plasma cells; and (4) BAFF/APRIL blockade can ameliorate immune-complex glomerular disease without completely disrupting humoral immunity. These concepts underpin the development of targeted anti-APRIL monoclonal antibodies and TACI-Fc fusion proteins currently in late-phase IgAN trials.

## Therapeutic targets of different B-cell modulators in IgAN

### Targeting mucosal immunity: nefecon

The hypothesis of mucosal origin of the pathogenic IgA1 was assessed in the NEFIGAN phase 2b trial (NCT01738035). Nefecon (a targeted-release formulation of budesonide) is designed to deliver high drug concentrations to the GALT present in the terminal ileum, where mucosal IgA synthesis in Peyer’s patches is particularly active, before undergoing extensive first-pass hepatic metabolism that thereby helps minimize systemic glucocorticoid effects. Treatment with Nefecon significantly reduced proteinuria in patients with IgAN compared with placebo. This supports the hypothesis that modulation of GALT-derived immune activity upstream of IgA immune-complex deposition could improve kidney outcomes, thereby validating gut-associated B cells as therapeutic targets in IgAN (107). These findings were further confirmed in the phase 3 NefIgArd trial (NCT03643965), which evaluated the efficacy and safety of safety of 9-month treatment of nefecon 16 mg/day 16 mg/day versus placebo in patients with IgAN with eGFR ≥ 35 mL/min/1.73 m^2^ and proteinuria ≥ 1 g/day despite at least 3 months of optimized supportive care. Treatment was followed by a 15-month observational period after discontinuation of the study drug. A total of 364 IgAN patients were enrolled in the study (nefecon 182, placebo 182). Majority were men (67.8%), and this was a predominantly White cohort (85.9%). Median age at enrollment was 43 years, median urine protein to creatinine ratio (UPCR) at baseline was 1.26 g/g, median total urine protein was 2.23 g/day (approximately 60% of patients had baseline proteinuria of ≥ 2 g/day), and the median eGFR was 55 mL/min/1.73 m^2^. Majority (65.3%) of patients had dipstick positive microhematuria at baseline. The median interval between biopsy-confirmed IgAN diagnosis and study enrollment was 2.5 years. At 2 years, nefecon demonstrated a significant treatment benefit compared with placebo (difference, 5.05 mL/min/1.73 m^2^), with a time-weighted average eGFR change of –2.47 mL/min/1.73 m^2^ with nefecon and –7.52 mL/min/1.73 m^2^ with placebo. A maximal 50% reduction in UPCR was observed in the nefecon group compared with placebo at 12 months. However, by the end of the 2-year observational follow-up, UPCR had begun to rise again, suggesting that a single 9-month course of nefecon is unlikely to provide a sustained clinical response (108). Studies exploring longer-term modified treatment protocols with nefecon are currently underway (NCT06712407). NefIgArd was the first phase 3 clinical trial to demonstrate significant improvements in both proteinuria and eGFR, reinforcing the disease-modifying potential of targeting mucosal B-cell activation, plasma cells, and the gut–kidney immune axis in IgAN (108, 109).

Mechanistically, these clinical benefits were further supported by biomarker analyses from the NEFIGAN and NefIgArd trials, which demonstrated that nefecon significantly reduced serum levels of FABP2 (Fatty Acid-Binding Protein 2, a marker of intestinal injury), systemic IgA antibodies to dietary antigens (gliadin and casein), BAFF, APRIL, secretory IgA, Gd-IgA1, and IgA-containing immune complexes (110). These findings suggest that nefecon acts upstream of glomerular injury by suppressing dysregulated GALT-driven B-cell and plasma-cell activity, supporting the notion that it is a disease-modifying therapy in IgAN (110). Nefecon was the first therapy to receive both accelerated and later full approval from the U.S. Food and Drug Administration (U.S. FDA) and the European Medicines Agency for the treatment of IgAN.

### B-cell depletion strategies (anti-CD20 agents)

#### Rituximab

Rituximab is a chimeric murine/human monoclonal antibody directed against CD20, a surface antigen expressed on B-cells. Rituximab is used for depletion of both normal and pathogenic B-cells while sparing plasma cells and hematopoietic stem cells that lack the CD20 surface antigen (111). In an open label trial, 34 adult IgAN patients with proteinuria ≥ 1 g/day were randomized to receive rituximab or standard conservative therapy. Treatment with rituximab depleted B-cells and was well tolerated; however, rituximab did not reduce proteinuria compared with baseline or the control group, and no change in eGFR was observed in either group. Serum levels of Gd-IgA1 and anti-Gd-IgA1 antibodies also remained unchanged (112). The reasons for this lack of efficacy remain unclear, but they may relate to rituximab’s inability to deplete CD20-negative plasmablasts, its limited penetration into kidney tissue, or the relative resistance of mucosal immune tissue to CD20 depletion because of high local levels of B-cell survival factors such as BAFF and APRIL within its microenvironment (113).

#### Obinutuzumab

Obinutuzumab is a glycoengineered, humanized, anti-CD20 monoclonal antibody that produces profound B-cell depletion. Obinutuzumab, also called type II anti-CD20 antibody, binds to CD20 proteins on B cells in a different way than rituximab. Type II anti-CD20 antibodies deplete B cells by induce programmed cell death and through enhanced antibody-dependent cell-mediated cytotoxicity. It has an enhanced affinity for the FcγIIIA receptor on B-cells through Fc-glycans modification, which can provide more effective and more sustained B-cell depletion than rituximab. Furthermore, it enhances antibody-dependent cellular cytotoxicity/phagocytosis and direct B-cell death compared with conventional type I anti-CD20 antibodies like rituximab (114). Clinical evidence regarding its utility in IgAN currently is extremely limited. The first published IgAN experience is a 2025 case series of three Chinese patients with biopsy-proven, progressive/refractory IgAN with persistent microscopic hematuria and nephrotic-range proteinuria despite prior favorable responses to supportive and immunosuppressive therapy (various combinations of glucocorticoids, mycophenolate mofetil, and cyclosporin). Mean time to administration of obinutuzumab was 2–4 years following prior treatments. Baseline proteinuria varied from 4.19-5.51 g/day, with baseline eGFR 41–84 mL/min/1.73 m^2^. Patients received intravenous obinutuzumab 1 g (1–2 doses); one patient had previously had an incomplete response to rituximab. After 12 months of treatment with obinutuzumab, all three patients had reduced proteinuria and hematuria, stable/improved kidney function, lower IgA/C3 ratio, and sustained B-cell depletion. Two patients had mild infusion reactions, and one had a mild upper respiratory infection; no serious infections or leukopenia were reported (115). Further and more rigorous data regarding the efficacy and safety of obinutuzumab in a global IgAN population is needed.

### BAFF/APRIL/Dual BAFF and APRIL axis targeting strategies

#### Anti-BAFF therapies

##### Blisibimod

The phase 1/2 BRIGHT-SC study (NCT02062684) evaluated the efficacy and safety of blisibimod, a selective BAFF inhibitor, in patients with IgAN (116). Preliminary findings presented in 2016 showed that treatment with blisibimod significantly reduced B-cell subsets and immunoglobulin levels, providing evidence of effective pharmacologic BAFF suppression (117). Proteinuria remained stable in the blisibimod-treated group, whereas the placebo group showed significant worsening. However, to date, the full study results have not been published, and the trial was later withdrawn. As a result, the long-term efficacy of therapies that selectively target BAFF alone in IgAN remains uncertain.

##### Belimumab

Belimumab is a fully human recombinant IgG1-λ monoclonal antibody that selectively binds to and inhibits BAFF, thereby reducing B-cell survival and activation (118). In a case report of a 14-year-old Chinese female with refractory crescentic IgAN despite utilization of multiple immunosuppressive medications in the past, subsequent belimumab administration led to proteinuria reduction and kidney function stabilization at 18 months. No adverse events including infection were noted (119).

A retrospective cohort study evaluated biopsy-proven IgAN patients treated at a single center in China between July 2020 and June 2024. Sixty-nine patients receiving belimumab were compared with 137 propensity-score-matched controls receiving standard therapy. Standard therapy included conservative management as well as prior immunosuppression use (oral glucocorticoids, hydroxychloroquine, leflunomide, mycophenolate mofetil, calcineurin inhibitors, cyclophosphamide, and combination therapies). The primary endpoint was proteinuria remission, defined as a ≥ 50% reduction in 24-hour urinary protein excretion or UPCR from baseline. Belimumab was administered intravenously at 10 mg/kg every 4 weeks. Among 206 patients (median age 37 years; baseline eGFR 79 mL/min/1.73 m^2^; proteinuria 1.1 g/day), 84% achieved proteinuria remission within 12 months. Remission rates were consistently higher with belimumab: 59% vs 46% at 3 months, 77% vs 66% at 6 months, and 93% vs 80% at 12 months (*p* = 0.025 at 12 months). Time-averaged UPCR was also lower in the belimumab group at 6 and 12 months. Among newly diagnosed patients, belimumab was associated with significantly higher remission rates at both 3 months (76.5% vs 47.1%, *p* = 0.009) and 6 months (88.2% vs 67.6%, *p* = 0.045). Similar or greater reductions in proteinuria were observed in patients with baseline UPCR > 600 mg/g and in those receiving renin-angiotensin-aldosterone-system inhibition. Importantly, belimumab was not associated with an increased incidence of common adverse events (120). Despite the positive findings, several limitations existed. This was a retrospective, single Chinese center-based study with a relatively small sample size, limited follow-up, with incomplete pathology data. Treatment decisions were physician-directed, and variations in concomitant therapies, including corticosteroid exposure, may have influenced kidney outcomes. Important immunologic biomarkers, including serum BAFF levels and B-cell phenotypes, were also not evaluated. These findings may not be directly generalizable to other global populations.

Larger, prospective, multicenter studies with longer follow-up and greater ethnic diversity are needed before belimumab can be considered as a potential IgAN treatment option around the world.

#### Anti-APRIL therapies

##### Sibeprenlimab [VIS649]

Sibeprenlimab is a fully humanized IgG2κ monoclonal antibody that selectively inhibits APRIL by preventing it from binding to its receptors (89, 121). In the phase 2 ENVISION trial [NCT04287985], intravenous sibeprenlimab administered every 4 weeks for 12 months in adult IgAN patients reduced proteinuria in a dose dependent manner, stabilized eGFR, suppressed serum levels of APRIL, and decreased levels of Gd-IgA1, with an acceptable safety profile (122).

In the phase 3 VISIONARY [NCT05248646] double-blind, randomized, placebo-controlled trial, adults with biopsy-proven primary IgAN and eGFR ≥ 30 mL/min/1.73 m^2^ with 24-hour UPCR ≥ 0.75 g/g or urine protein ≥ 1.0 g/day despite 3 months of maximal conservative therapy were randomized in a 1:1 ratio to receive either subcutaneous sibeprenlimab at a dose of 400 mg or placebo administered every 4 weeks for 100 weeks. The primary efficacy endpoint is to evaluate the change in 24-hour UCPR at 9 months compared with baseline, and the secondary endpoint is to evaluate the annualized slope of eGFR estimated over 100 weeks (123). A prespecified interim analysis of the VISIONARY study was performed at 9 months and included the first 320 amongst 510 study patients (152 received sibeprenlimab; 168 received placebo). At baseline, 62.5% of patients were men, 59.1% were Asian, median age was 42 years, mean eGFR was 63.4 mL/minute/1.73 m^2^, and median 24-hour UPCR was 1.25 g/g. The median time from initial kidney biopsy to randomization was 1.5 years. The trial met its primary outcome, with a significant reduction in 24-hour UCPR after 9 months of therapy with sibeprenlimab (–50.2%) while there was an increase with placebo (2.1%), corresponding to an adjusted geometric least-squares mean 24-hour UPCR of 51.2% (96.5% confidence interval [CI], 42.9 to 58.2) in favor of sibeprenlimab (*p* < 0.001). APRIL and Gd-IgA1 levels were reduced by 95.8% and 67.1%, respectively, with sibeprenlimab. Prevalence of microscopic hematuria was also significantly reduced in the sibeprenlimab group when compared to placebo. Sibeprenlimab was well tolerated with a safety profile comparable to placebo. In the sibeprenlimab-treated group, total IgA and IgM levels were reduced by approximately 70%, and IgG levels by 35%. Nine patients in the sibeprenlimab group had post-baseline IgG levels ≤ 400 mg/dL. No deaths were reported, and the incidence of serious adverse events that occurred during the treatment period was 3.5% with sibeprenlimab and 4.4% with placebo (124). Based on these favorable results, in November 2025 the U.S. FDA granted accelerated approval to sibeprenlimab (Voyxact) for reduction of proteinuria in adults with primary IgAN at risk for disease progression (125).

Updated data from August 2026 showed the VISIONARY study achieved its key secondary endpoint, with an annualized eGFR slope of +0.3 (95% CI, -0.4 to 0.9) mL/min/1.73 m^2^ per year with sibeprenlimab versus -4.2 (95% CI, -4.9 to -3.6) mL/min/1.73 m^2^ per year with placebo, demonstrating a statistically significant treatment effect of +4.5 (95% CI, 3.6 to 5.4; *p* < 0.0001) mL/min/1.73 m^2^ per year at two years (24 months). The annualized eGFR slope (the study’s key secondary endpoint) was estimated using a prespecified linear mixed-effects model that evaluated repeated eGFR measurements from week 4 through week 104 (24 months) to estimate the annual rate of kidney function change. At two years (24 months), the least-square-mean change from baseline in eGFR showed +1.3 mL/min/1.73 m^2^ with sibeprenlimab (95% CI, −0.3 to 2.8), compared with −7.9 mL/min/1.73 m^2^ with placebo (95% CI, −9.5 to −6.4), for a treatment difference of +9.2 mL/min/1.73 m^2^ (95% CI, 7.1 to 11.4; *p* < 0.0001). The overall safety profile of sibeprenlimab remained consistent throughout two years (24 months) and was comparable to placebo, with similar rates of overall adverse events (90.7% vs 90.0%), infections and infestations (51.4% vs 51.0%), and injection site reactions (35.1% vs 32.2%). No new safety signals were identified, and rates of serious infections were numerically lower with sibeprenlimab than placebo (1.9% vs 4.0%), alongside lower rates of serious and severe adverse events and treatment discontinuations (126, 127). A complete analysis of the Phase 3 VISIONARY two-year (24-month) results are awaited.

In the VISIONARY study, 88 of 256 (34%) patients in the treatment arm developed anti-drug antibodies (ADA) of which 21 (23.9%) developed ADA with neutralizing activity. In those patients with ADA, sibeprenlimab exposure was ~40% lower than in patients with undetectable ADA over the treatment period. The reduction in 24-hour UPCR from baseline to Month 9 was 41.6% vs. 52.7% in those who developed ADA and those who did not respectively (128). The clinical significance of these numerical differences in proteinuria reduction is not clear.

##### Zigakibart [BION-1301]

Zigakibart is a humanized IgG4 monoclonal antibody that binds to and blocks APRIL binding to BCMA and TACI. In an ongoing Phase 1/2 clinical trial that included 40 patients with IgAN [NCT03945318], treatment with intravenous zigakibart was well tolerated and resulted in robust and durable reductions in Gd-IgA1 levels and proteinuria, decreased microscopic hematuria, and ensured sustained stabilization of eGFR, which were maintained over 100 weeks of treatment (129). A phase 3 trial examining the benefit of zigakibart in IgAN is ongoing [BEYOND study, NCT NCT05852938] (130).

#### Dual BAFF and APRIL inhibitors

##### Atacicept

Atacicept is a first-in-class, fully humanized fusion protein consisting of the extracellular domain of TACI (a receptor for both BAFF and APRIL), fused to the Fc portion of IgG1, thereby creating a soluble receptor with potent affinity for both BAFF and APRIL cytokines (131). In the phase 2b ORIGIN trial, patients with IgAN were randomized to receive once-weekly subcutaneous atacicept at varying doses or placebo. Proteinuria reduced significantly in the atacicept group at weeks 24 and 36 when compared to placebo. Atacicept treatment was associated with eGFR stabilization compared to a decline with placebo at week 36. Gd-IgA1 decreased from baseline by 60% and remained relatively stable in placebo group. The safety profile of atacicept and placebo were similar with no major adverse events (132). Participants from the phase 2b ORIGIN trial were subsequently enrolled in an open-label extension study, in which all participants received subcutaneous atacicept 150 mg once weekly for an additional 60 weeks. Serum IgG, IgA, and IgM levels remained stable during the open-label extension period following initial reductions. Sustained decreases in Gd-IgA1, UPCR, and microscopic hematuria were observed. The mean annualized eGFR slope was –0.6 ± 0.5 mL/min/1.73 m^2^ over 96 weeks. Notably, participants who initially received placebo during the double-blind phase showed significant reductions in Gd-IgA1, microscopic hematuria, and UPCR after transitioning to atacicept 150 mg at week 36, comparable to those originally assigned to atacicept. This transition was also associated with an improvement in the eGFR trajectory (133).

The ORIGIN 3 [NCT04716231] is an ongoing, phase 3, multicenter, double-blind, randomized, placebo-controlled trial involving adult patients with kidney biopsy proven IgAN with 24-hour UPCR ≥ 1g/g or 24-hour proteinuria ≥ 1 g/day, eGFR ≥ 30 mL/min/1.73 m^2^, and stable conservative treatment ≥ 12 weeks. Patients were randomized in a 1:1 ratio to receive subcutaneous atacicept 150 mg once weekly versus placebo. The primary end point was the percentage change from baseline in the 24-hour UPCR at week 36 (134). Secondary outcome involves the annualized change in eGFR at 104 weeks. In a prespecified interim analysis of the ORIGIN 3 trial, 203 patients with IgAN were included, with 106 assigned to the atacicept group and 97 to the placebo group. For this interim analysis, the primary efficacy endpoint was the change in 24-hour UPCR, while secondary endpoints included changes in Gd-IgA1 levels and resolution of hematuria. Baseline demographic and clinical characteristics were comparable between the two groups. Mean age was 41 years, mean eGFR was 65 mL/min/1.73 m^2^, and median 24-hour UPCR was 1.8 g/g. The median interval from initial kidney biopsy to randomization was 2.5 years. Nearly all participants were receiving maximally tolerated doses of renin-angiotensin-aldosterone system inhibitors at enrollment, and notably, 53.2% were also on a stable dose of an SGLT2 inhibitor. At week 36, the 24-hour UPCR had decreased by 45.7% from baseline in the atacicept group, compared with 6.8% in the placebo group, yielding a geometric mean between-group difference of 41.8% (95% CI, 28.9-52.3; *p* < 0.001). Adverse events occurred in 59.3% of patients in the atacicept group and 50.0% of those in the placebo group, with most events being mild to moderate in severity. Treatment with atacicept resulted in reductions in serum IgG, IgA, and IgM levels, which then stabilized after week 24. Severe hypogammaglobulinemia, defined by serum IgG < 300 mg/dL, was not observed. Treatment with atacicept was also associated with significant reductions in Gd-IgA1 levels (68.3% in atacicept group versus 2.9% in the placebo group) and more frequent resolution of microscopic hematuria. Reductions in Gd-IgA1 levels occurred early in the trial, at 4 weeks, preceding the reduction in proteinuria noted at 12 weeks, which suggests that early immunological response translates to subsequent clinical benefit in patients on treatment with atacicept for IgAN (135). Based on these favorable results, in July 2026 the U.S. FDA granted accelerated approval to atacicept (Trutakna^®^) for reduction of proteinuria in adults with primary IgAN at risk for disease progression (136, 137). The trial is ongoing to evaluate the change in eGFR over a 2-year period and is expected to complete in late 2026.

In a pooled analysis of the phase 2 and phase 3 studies through week 36 in patients with primary IgAN, 43.2% (60/139) of participants treated with once-weekly atacicept developed ADAs. Despite this relatively high incidence of immunogenicity, ADA development had no clinically meaningful impact on the pharmacokinetics, pharmacodynamics, safety, or efficacy of atacicept over the 36-week treatment period (138). Neutralizing-antibody incidence has not been reported so far. Long term data on ADAs for atacicept is awaited.

##### Povetacicept

Povetacicept is an engineered variant of TACI (transmembrane activator and CAML interactor) receptor linked to an Fc region of IgG and produced as a recombinant fusion protein designed to achieve complete inhibition of the cytokines BAFF and APRIL (139). The RUBY-3 trial [NCT05732402] is an open-label phase 1b/2a study evaluating povetacicept in adults with various glomerular diseases, including IgAN (140). For participants with IgAN, eligibility required a kidney biopsy performed within the preceding 10 years, eGFR ≥ 30 mL/min/1.73 m^2^, and proteinuria ≥ 0.5 g/g. Povetacicept was administered subcutaneously every 4 weeks to 54 participants with IgAN, with 21 receiving the 80 mg dose and 33 receiving the 240 mg dose. In an interim analysis, participants with IgAN on 80 mg had a 64% mean 24-hour UPCR decrease (95% CI –76 to –48) from baseline with 65% of participants achieving UPCR < 0.5 g/g and stable eGFR at week 48. Early decline in Gd-IgA1 noted week 12 (57%), continued through week 48 (77%). Around 90% of participants achieved hematuria resolution and 53% achieved clinical remission (defined as 24-hr UPCR<0.5 g/g, negative hematuria and eGFR reduction of <25% from baseline). Efficacy outcomes were similar with 240 mg povetacicept (141).

RAINIER [NCT06564142] is a global phase 3, randomized, double-blind, placebo-controlled trial evaluating subcutaneous povetacicept 80 mg administered every 4 weeks versus placebo in patients with IgAN, with proteinuria reduction and eGFR as key outcomes (142). In the main cohort of 557 patients, the median time from IgAN diagnosis to randomization was approximately 3.8 years, 97.8% were receiving renin-angiotensin aldosterone inhibitors and 67.7% receiving SGLT2 inhibitors likely reflective of the change in clinical practice intensifying background supportive care. In a recently announced interim analysis of 199 patients (131 receiving povetacicept and 68 receiving placebo), treatment with povetacicept resulted in a 52% reduction in UPCR from baseline at week 36 and a statistically significant, clinically meaningful 49.8% greater UPCR reduction compared with placebo (*p* < 0.0001). The trial also met its secondary endpoints. Serum Gd-IgA1 dropped by 77.4% from baseline in patients treated with povetacicept whereas it increased by 9.1% in the placebo resulting in a 79.3% reduction relative to placebo (*p* < 0.0001). Among participants who had hematuria at baseline, 85.1% achieved resolution of hematuria with povetacicept treatment, compared with 23.4% in the placebo group, corresponding to a 61.7% greater rate of hematuria resolution with povetacicept (*p* < 0.0001). Povetacicept was generally safe and well tolerated, with most adverse events being mild to moderate in severity. No serious adverse events were attributed to povetacicept, and no deaths occurred during the trial. There were also no opportunistic infections and no treatment discontinuations due to infections. Although anti-drug antibodies were detected, they did not appear to affect efficacy or safety (143). The peer-review publication of results from this trial is awaited.

##### Telitacicept

Telitacicept is a recombinant fusion protein comprising the extracellular domain of human TACI linked to the Fc domain of human IgG that inhibits both BAFF and APRIL cytokines and has shown efficacy in clinical and real-world studies from China (144, 145). Non-randomized real-world studies have also demonstrated reductions in proteinuria and stabilization of kidney function in patients with IgAN (145–151). Telitacicept has also been shown to reduce both circulating Gd-IgA1 levels and IgA-containing immune complexes (152).

The phase 3, multicenter, randomized, double-blind, placebo-controlled TELIGAN study conducted in two prespecified parts (stages A and B) enrolled adult patients with primary IgAN randomized to receiving weekly telitacicept versus placebo. This trial was done in China. Stage A evaluated the effect of telitacicept on proteinuria over 39 weeks, while the ongoing Stage B is assessing its longer-term impact on kidney function through up to 104 weeks of treatment. The study enrolled Chinese adults with biopsy-proven primary IgA nephropathy (IgAN), persistent proteinuria defined as a 24-hour UPCR ≥ 0.8 g/g or 24-hour urinary protein ≥ 1 g/day, and an eGFR ≥ 30 mL/min/1.73 m^2^ despite optimized supportive care. Participants were randomized 1:1 to receive subcutaneous telitacicept 240 mg once weekly or matching placebo. The primary endpoint of Stage A was the geometric mean ratio of 24-hour UPCR at week 39 relative to baseline, with safety assessed throughout the study. A prespecified interim analysis of Stage A has been reported (153). A total of 318 patients were randomized to receive telitacicept or placebo, with 159 participants in each group. The mean age was 38.2 years, and 53.8% of participants were women. Baseline kidney function was comparable between groups, with a mean eGFR of 76.53 ± 26.95 mL/min/1.73 m^2^ in the telitacicept arm and 74.43 ± 27.20 mL/min/1.73 m^2^ in the placebo arm. Median 24-hour UPCR was 1.23 g/g (range, 0.47-5.86) with telitacicept and 1.29 g/g (range, 0.54-6.59) with placebo. All participants were receiving renin-angiotensin-aldosterone system inhibitors, and approximately 35% were also treated with sodium-glucose cotransporter 2 (SGLT2) inhibitors. At week 39, patients in the telitacicept arm had a substantial reduction in proteinuria, with a 58.9% decrease in 24-hour UPCR from baseline compared with an 8.8% reduction with placebo. This corresponded to a 55.0% relative reduction in UPCR versus placebo (95% CI, −61.3 to −47.6; *p* < 0.001). Separation between treatment groups was evident as early as week 4 and progressively widened through week 39, with consistent proteinuria benefits across all prespecified subgroups. Kidney function was also better preserved with telitacicept. The mean percentage change in eGFR from baseline was −1.0% (95% CI, −3.2 to 1.2) with telitacicept compared with −7.7% (95% CI, −9.9 to −5.4) with placebo. A confirmed ≥ 30% decline in eGFR occurred in 6.3% of patients receiving telitacicept versus 27.0% receiving placebo, suggesting a potentially favorable effect on preservation of kidney function. *Post hoc* analyses demonstrated additional immunologic and clinical effects of telitacicept. The proportion of patients with hematuria decreased from 71% at baseline to 21% at week 39 in the telitacicept group, while remaining largely unchanged with placebo. Circulating CD19+ B-cell levels declined by 46.4% with telitacicept compared with a 4.5% increase with placebo. Serum immunoglobulin levels also progressively decreased during treatment; at week 39, serum IgA, IgG, and IgM levels were reduced by 60.6%, 13.2%, and 78.6%, respectively, relative to placebo. Importantly, no patients developed severe hypogammaglobulinemia, defined as a serum IgG level ≤ 3 g/L. Adverse events were more frequent with telitacicept than with placebo (89.3% vs 78.6%), although most were mild to moderate and were primarily related to injection-site reactions. Serious adverse events were less common with telitacicept (2.5% vs 8.2%), and no unexpected safety signals or deaths were reported. Among 68 patients with latent hepatitis B virus (HBV) infection at baseline, none of the 31 patients receiving telitacicept experienced HBV DNA reactivation during the 39-week treatment period (153). Stage B of the study, assessing effect of treatment on eGFR at 104 weeks, is currently ongoing.

Although these studies suggest that telitacicept may be an effective and well-tolerated treatment for IgAN in Chinese patients, additional data in non-Asian populations, particularly non-Chinese patients, are needed.

### Plasma cell-directed therapies

#### CD38-targeting therapies

CD38 is expressed on long-lived plasma cells, which may contribute to the overproduction of pathogenic Gd-IgA1 and thereby promote the formation of Gd-IgA1–containing immune complexes. By targeting and depleting CD38+ plasma cells, CD38 inhibitors are intended to reduce Gd-IgA1-driven immune activity and help limit kidney injury (154). Anti-CD38 monoclonal antibodies bind to plasma cells and induce apoptosis and cell death through mechanisms including antibody-dependent cell-mediated cytotoxicity by natural killer cells and antibody-dependent cellular phagocytosis by macrophages. In addition, anti-CD38 monoclonal antibodies can induce complement-mediated cytotoxicity. These agents are already used clinically for the treatment of multiple myeloma, and their use has previously been associated with greater reductions in polyclonal IgA than in IgG (155).

##### Felzartamab

Felzartamab is a fully human IgG1 anti-CD38 monoclonal antibody designed to deplete CD38+ plasma cells, which are considered a potential source of pathogenic Gd-IgA1 and autoantibodies in IgAN (156). The phase 2a IGNAZ study [NCT05065970], a randomized, double-blind, placebo-controlled trial, evaluated the efficacy and safety of intravenous felzartamab compared with placebo in adults with IgAN. Eligible patients had biopsy-confirmed IgAN, proteinuria ≥ 1.0 g/day (or ≥ 0.5 g/day for Japanese patients), eGFR ≥ 30 mL/min/1.73 m^2^ and had been receiving renin-angiotensin-aldosterone system inhibitors for at least 3 months. Among the 54 patients in the overall cohort, 67% were men, mean age was 41.6 years, mean UPCR was 1.7 g/g, and mean eGFR was 74.6 mL/min/1.73 m^2^. In part 1, patients were randomized during the 6-month treatment period to receive either intravenous placebo (12 patients) or felzartamab across three dosing regimens: two doses over 15 days (12 patients), five doses over 2 months (11 patients), or nine doses over 5 months (13 patients). In part 2, six Japanese patients received the 9-dose felzartamab regimen in an open-label setting. Follow-up extended to 24 months in part 1 and 12 months in part 2, measured from the first dose. The primary endpoint was the change in UPCR at 9 months. Participants receiving felzartamab experienced rapid and clinically meaningful reductions in UPCR that were sustained through month 9, with the greatest effect observed in the 9-dose arm (−44.5%), compared with those receiving placebo. Mean rates of eGFR loss were smaller with felzartamab than with placebo through 24 months in part 1 and 12 months in part 2. In the felzartamab treatment arms, reductions in IgA were rapid and persisted for up to 19 months after the final dose. IgG levels on the other hand recovered within six to nine months. Overall, treatment-emergent adverse events were generally grade 1 or 2 and showed no dose dependence. Infusion-related reactions were the most common treatment-related adverse event and typically occurred with the first dose (157). The phase 3 PREVAIL trial [NCT06935357] is currently recruiting globally to ascertain the effects of felzartamab on proteinuria reduction and effect on eGFR in IgAN (158).

##### Mezagitamab (TAK-079)

Mezagitamab is another fully humanized IgG1 anti-CD38 monoclonal antibody that depletes plasma cells (159). In an open-label, single-arm, phase 1b trial (NCT05174221) (160), 17 participants with IgAN enrolled (mean age 40.8 years, 53% female, and 71% Asian). At study entry, mean disease duration was 4.6 years, UPCR was 1.5 g/g, and eGFR was 74.0 mL/min/1.73 m^2^. Mezagitamab was administered intravenously (16 doses over 24 weeks) and was generally well tolerated without any opportunistic infections or grade 3 or higher infections. There were rapid and sustained reductions in IgA (nadir, −70.1%) and Gd-IgA1 (nadir, −62.2%), accompanied by a 54.9% reduction in proteinuria and stable kidney function at week 36 (161). A phase 3 global trial [NCT06963827] is currently recruiting to ascertain the effects of mezagitamab on proteinuria reduction and effect on eGFR in IgAN (162).

The effects of anti-CD38 monoclonal antibodies on lowering IgA, Gd-IgA1, and proteinuria appear to persist for several months, potentially supporting intermittent dosing strategies as clinically needed. A summary of B-cell and plasma-cell targeted therapies in phase 3 clinical studies (**Figure 2**) currently in development for IgAN are summarized (**Table 1**).

**Table 1:** B-Cell and plasma cell-targeted therapies currently in development for IgAN.

Agent	Molecule type	Mechanism of action	Identifier	Clinical trial	Main outcome measures	Proteinuria reduction (%)
Sibeprenlimab	Humanized IgG2κ mAb	Selective APRIL inhibition	NCT05248646	VISIONARY (Phase 3)	Change in 24-hr UPCR at 9 mo. Annualized eGFR slope at 24 mo.	−51.2% vs placebo for proteinuria +4.5 mL/min/1.73m^2^/yr. vs placebo
Zigakibart	Humanized IgG4 mAb	Selective APRIL inhibition	NCT05852938	BEYOND (Phase 3, ongoing)	Change in UPCR from baseline at week 40 Annualized change in eGFR from baseline to week 104	Not reported yet
Atacicept	TACI–Fc fusion protein	Dual BAFF and APRIL inhibition	NCT04716231	ORIGIN 3 (Phase 3, interim analysis)	% change in the 24 hr-UPCR from baseline Annualized rate of change in eGFR through week 104	−45.7% reduction in proteinuria from baseline
Povetacicept	TACI–Fc fusion protein	Dual BAFF and APRIL inhibition	NCT06564142	RAINIER (Phase 3, ongoing)	Reduction in 24-hr UPCR from baseline at week 36 Total change in eGFR slope through week 104	-52% reduction in UPCR from baseline compared to -4.4% in placebo group
Telitacicept	TACI–Fc fusion protein	Dual BAFF and APRIL inhibition	NCT05799287	TELIGAN (Phase 3, data from stage A)	Change in 24-hr UPCR at week 39 Total slope decline in eGFR through week 104	−58.9% reduction in UPCR compared to -8.8% in placebo group
Felzartamab	Humanized anti-CD38 mAb	CD38^+^ plasma-cell depletion	NCT06935357	PREVAIL (Phase 3, recruiting)	Percentage change from baseline in 24-hr UPCR at week 36 Change in eGFR from baseline to week 104	Not reported yet
Mezagitamab	Humanized anti-CD38 mAb	CD38^+^ plasma-cell depletion	NCT06963827	AMIGA (Phase 3 recruiting)	Change from baseline in 24-hr UPCR at week 36 Change from baseline in eGFR at week 52 and 104	Not reported yet

IgAN, Immunoglobulin A nephropathy; UPCR, urine protein-to-creatinine ratio; eGFR, estimated glomerular filtration rate; APRIL, a proliferation-inducing ligand; BAFF, B-cell activating factor; BCMA, B-cell maturation antigen; mAb, monoclonal antibody; TACI, transmembrane-activator-and-calcium-modulator-and-cyclophilin-ligand-interactor.

#### Proteasome inhibitors

Proteasome inhibition represents an alternative intracellular strategy for targeting long-lived plasma cells. In small IgAN cohorts, intravenous bortezomib, a proteasome inhibitor, has been associated with reductions in proteinuria (163, 164). However, further studies are needed to evaluate its effects on kidney function stabilization and overall safety in this population.

### Targeting intracellular B-cell signaling pathways

Targeting intracellular signaling pathways in B-cells represents an alternative approach to interrupting the production of pathogenic Gd-IgA1 and the downstream inflammatory processes that drive IgAN. This strategy includes inhibition of kinases involved in B-cell receptor (BCR) signaling. Fostamatinib, an oral spleen tyrosine kinase (SYK) inhibitor, failed to produce a significant reduction in proteinuria in IgAN patients at 24 weeks in a phase 2 trial and was therefore not pursued further (165).

Several selective Bruton’s tyrosine kinase (BTK) inhibitors have advanced into clinical development for autoimmune diseases by targeting downstream BCR signaling. These include covalent inhibitors such as evobrutinib and tolebrutinib, both of which have progressed to Phase 3 trials, as well as the reversible, non-covalent inhibitor fenebrutinib. Across multiple autoimmune indications, these agents have demonstrated promising pharmacodynamic activity (166). Early BTK inhibitors, such as spebrutinib (NCT04599917), were evaluated in clinical trials for autoimmune diseases including rheumatoid arthritis, providing initial evidence for the therapeutic potential of BTK inhibition in immune-mediated disorders. However, further clinical development of spebrutinib was subsequently discontinued.

TNF receptor-associated factor (TRAF) is an important linker molecule in the TNF superfamily; TRAF6 is involved in inflammation and immune responses by mediating inflammatory and apoptotic signaling pathways (167). In addition, TRAF may serve a key role by activating the nuclear factor κB (NF-κB) signaling pathway, that is an important intracellular inflammatory transcription pathway (168). Activation of the TRAF6/NF-κB signaling pathway enhances the production of profibrotic mediators in glomerular mesangial cells, while the BAFF receptor Fc chimeric protein suppresses this signaling pathway (169). Further research is needed on the BAFF/APRIL axis and NF-κB in IgAN before its clinical implementation in IgAN can be pursued.

### Biomarkers in IgAN

In recent years, the identification of several novel biomarkers has created new opportunities for more personalized approaches to IgAN. Promising candidates include galactose-deficient IgA1 (Gd-IgA1) and corresponding autoantibodies, immune-complex assays, complement related markers, BAFF/APRIL levels, plasma-cell signatures, mucosal immune markers, various urinary markers including urinary CD 163, MMP-7 and KIM-1, and microRNAs such as miR-148b and let-7b that hold potential for diagnosis and prognosis assessment (170, 171). As these data accumulate, meta-analyses may help validate the prognostic values of emerging biomarkers and their ability to predict treatment response, while mediation analyses could clarify whether biomarker changes directly contribute to improvements in clinical outcomes. Ultimately, rigorous validation and assay standardization across diverse populations could support the incorporation of novel biomarkers into clinical practice to optimize personalized patient management. Existing risk-stratification models combining clinical parameters and histopathologic features may in the future integrate molecular biomarkers such as Gd-IgA1, miRNAs, and complement profiles to improve prediction of disease progression. Additionally, novel biomarkers could serve regulatory frameworks as surrogate endpoints for kidney outcomes or eGFR decline, potentially accelerating therapeutic development and approval, similar to the established use of proteinuria (171, 172).

## Conclusion and future directions

B-cell-targeting therapies, including those targeting the BAFF and APRIL pathways, offer an immunologically pertinent strategy for the treatment of IgAN, a condition associated with immune dysregulation with scientific evidence supporting the central role of BAFF/APRIL axis and long-lived plasma cells. Emerging early- and late-phase clinical trials support this biological plausibility with selective APRIL inhibition, dual BAFF/APRIL blockade, and plasma cell-directed therapies showing reduction in proteinuria, hematuria, pathogenic Gd-IgA1, and preservation of kidney function. Importantly these novel therapies have so far been generally well tolerated with reassuring safety profiles in the medium term.

Despite the rapidly evolving therapeutic landscape in IgAN, several important questions remain unresolved. How will BAFF-APRIL and plasma cell targeted therapies be incorporated into an increasingly complex treatment algorithm? Whether combination strategies will be used and in whom? This would include immunosuppressive with non-immunosuppressive treatments, but also the potential need for multiple immunologic therapies in patients with particularly aggressive phenotypes. Such strategies may be evaluated formally in future prospective trials but may also emerge from real-world evidence. Similarly, whether dual BAFF/APRIL inhibition offers meaningful mechanistic or clinical advantages over selective APRIL blockade remains uncertain in the absence of head-to-head comparison studies. Another emerging concept is whether we should consider treating IgAN patients with an “induction” followed by a “maintenance” phase to prevent further kidney injury. Whether baseline serologic, urinary, or tissue biomarkers could help identify patients more likely to benefit from one approach over the other is another clinically important unanswered question that might be addressed by ongoing mechanistic studies.

An updated 2026 KDIGO commentary to the previous 2025 guideline pertaining to IgAN management has included APRIL inhibitors and dual BAFF-APRIL inhibitors in the list of agents that manage the IgAN-specific drivers for nephron loss (173). One thing that is clear is that B-cell-targeting therapies should be instituted promptly in patients at high risk of disease progression.

Finally, the frequency of use to maintain stable kidney function, durability of therapeutic responses, longer-term safety, clinical outcomes following treatment discontinuation remain major knowledge gaps. Ongoing phase 3 trials and their open-label extension studies should provide important insights into these issues over the coming years, as will real world evidence. Regardless, we are witnessing an exciting, hopeful paradigm shift in the treatment of IgAN and heralding a new era for immune-mediated glomerular diseases more broadly.
